# Supplementary material for: Selective sonochemical post-synthesis modification of LTA zeolite with zinc species
Source: PLoS One. 2025 Jun 20;20(6):e0324997. doi: 10.1371/journal.pone.0324997 (PMC12180657; doi:10.1371/journal.pone.0324997)
Supplement: S1 Table — Statistical parameters for the obtained models from the N2 adsorption-desorption isotherms. (DOCX) [file pone.0324997.s001.docx]

Table S1: Statistical parameters for the obtained models from the N_2_ adsorption-desorption isotherms.

|  | Langmuir | | | | | t-plot | | | | | NLDFT |
| --- | --- | --- | --- | --- | --- | --- | --- | --- | --- | --- | --- |
|  | Slope | ± | Intercept | ± | r | Slope | ± | Intercept | ± | r | STD DEV |
| NaA | 3.038289 | 0.32186 | 10.6018 | 3.5034 | 0.973073 |  |  |  |  |  | 0.41922 |
| [Zn(OH)2@NaA](mailto:NaA@Zn(OH)2) | 1265.37 |  | 30.48 |  | 0.976 |  |  |  |  |  | 0.3798 |
| [ZnO@NaA](mailto:NaA@ZnO) | 0.00994 | 0.000079 | 0.015 | 0.0018 | 0.999968 | 51.334548 | 1.014147 | 71.28735 | 0.431385 | 0.998248 | 1.89452 |
| ZnO_2_@NaA | 0.00748966 | 0.000011 | 0.0033 | 0.0002 | 0.999998 | 7.316308 | 0.042539 | 128.78415 | 0.034811 | 0.99973 | 2.44826 |
| r= correlation coefficient, STD DEV= standard deviation of the fit (cm3/g STP) | | | | | | | | | | |  |
