# Supplementary material for: Selective sonochemical post-synthesis modification of LTA zeolite with zinc species
Source: PLoS One. 2025 Jun 20;20(6):e0324997. doi: 10.1371/journal.pone.0324997 (PMC12180657; doi:10.1371/journal.pone.0324997)
Supplement: S1 Fig — A) Speciation of Zn at a concentration of 10 μM at pH = 8 for obtaining Zn(OH)2 and B) 0.10 mM at pH 10 for ZnO. (DOCX) [file pone.0324997.s002.docx]

**
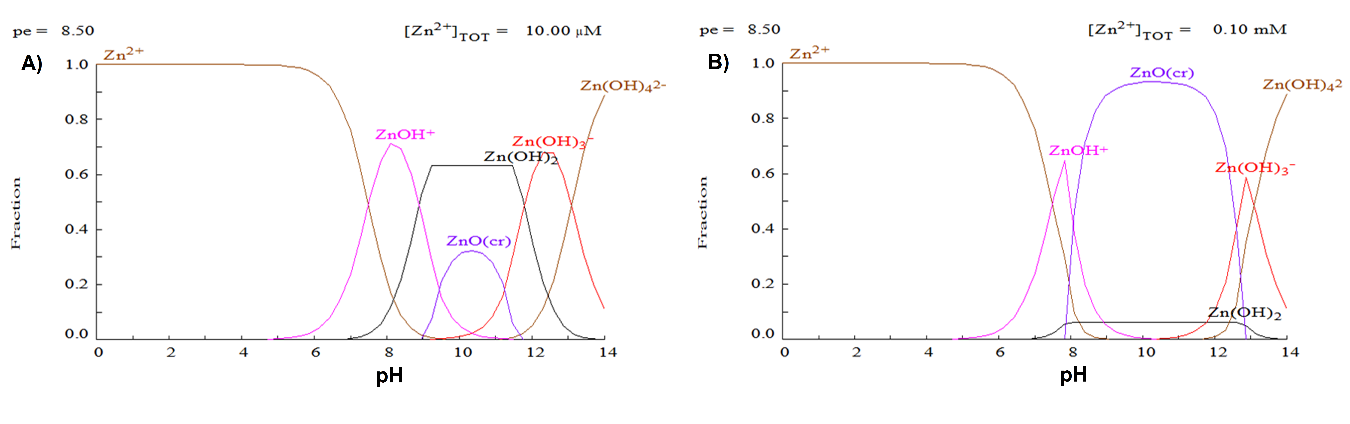
**

**S1 Fig. Speciation diagrams**. A) Speciation of Zn at a concentration of 10 μM at pH=8 for obtaining Zn(OH)_2_ and B) 0.10 mM at pH 10 for ZnO.
