# Supplementary material for: Selective sonochemical post-synthesis modification of LTA zeolite with zinc species
Source: PLoS One. 2025 Jun 20;20(6):e0324997. doi: 10.1371/journal.pone.0324997 (PMC12180657; doi:10.1371/journal.pone.0324997)
Supplement: S2 Fig — Difractograms of Zeolite NaA treated at the ultrasonic conditions to generate ZnO2, (O2)@NaA, and Zn(OH)2, (OH)@NaA, without the addition of Zn precursors. (DOCX) [file pone.0324997.s003.docx]

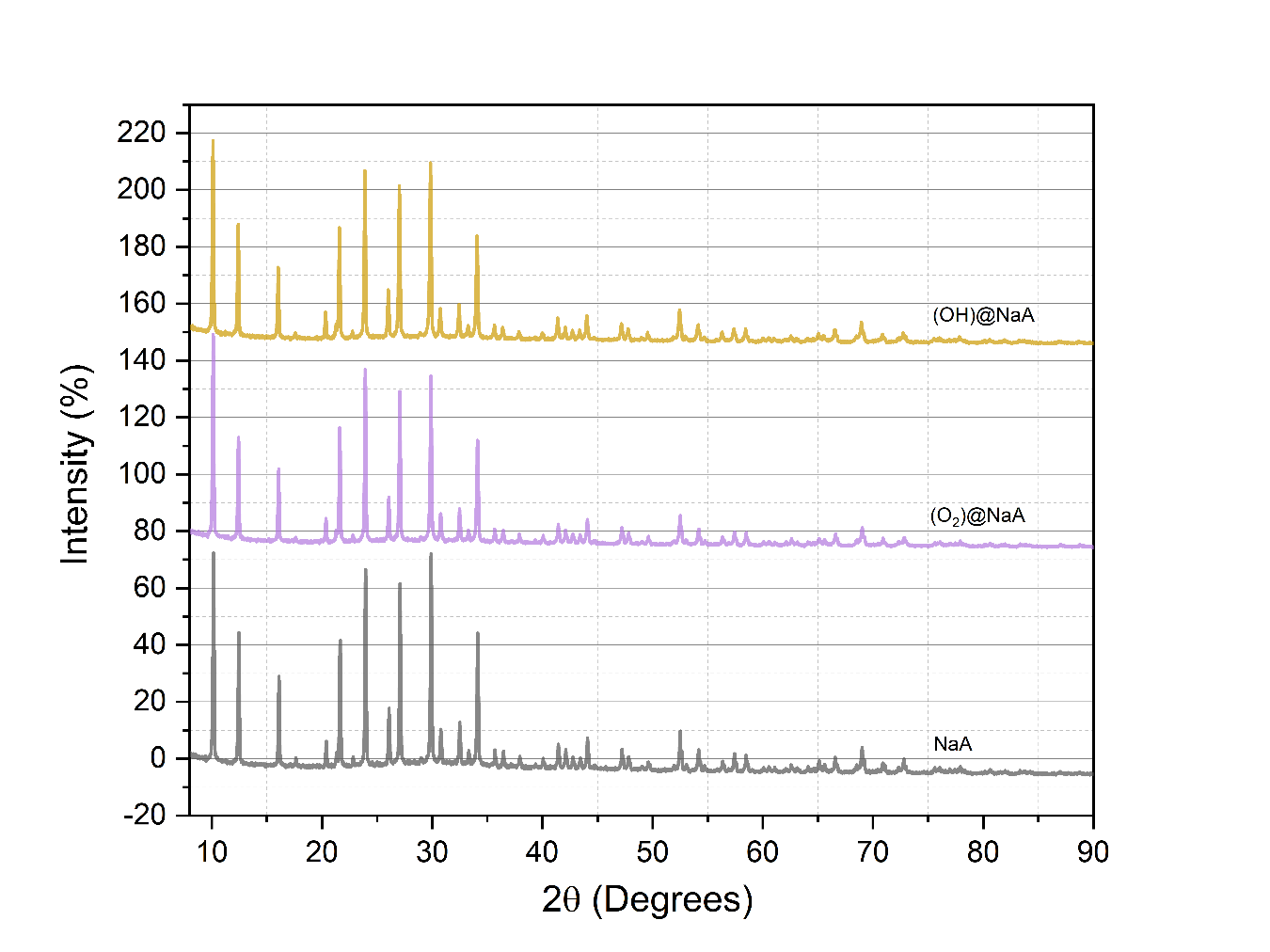


**S2 Fig. X-ray diffraction measurements.** Difractograms of Zeolite NaA treated at the ultrasonic conditions to generate ZnO_2_, (O_2_)@NaA, and Zn(OH)_2_, (OH)@NaA, without the addition of Zn precursors.
