# Supplementary material for: Selective sonochemical post-synthesis modification of LTA zeolite with zinc species
Source: PLoS One. 2025 Jun 20;20(6):e0324997. doi: 10.1371/journal.pone.0324997 (PMC12180657; doi:10.1371/journal.pone.0324997)
Supplement: S3 Fig — Replicates for the isotherms of zeolite NaA after excessive washing and drying. (DOCX) [file pone.0324997.s004.docx]

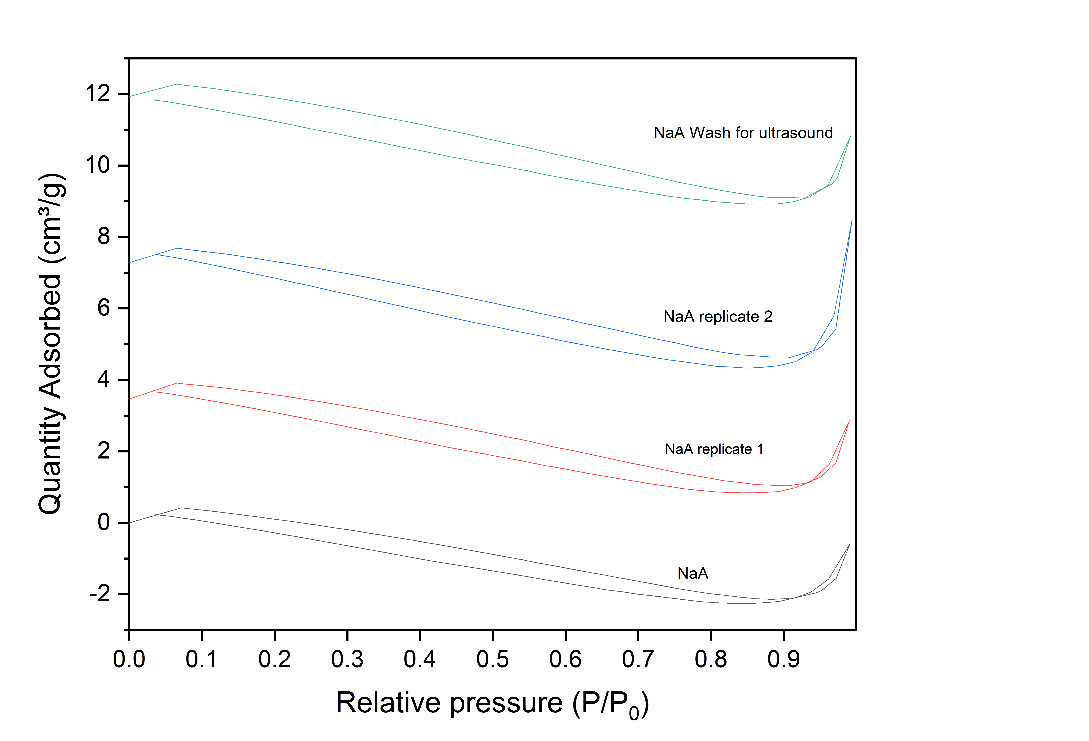


**S3 Fig. N_2_ Adsorption-desorption isotherms.** Replicates for the isotherms of zeolite NaA after excessive washing and drying.
