# Supplementary material for: Selective sonochemical post-synthesis modification of LTA zeolite with zinc species
Source: PLoS One. 2025 Jun 20;20(6):e0324997. doi: 10.1371/journal.pone.0324997 (PMC12180657; doi:10.1371/journal.pone.0324997)
Supplement: Attachment — Submitted filename: Final Response to ReviewersPONE_D_25_12190.pdf [file pone.0324997.s007.pdf]

PONE-D-25-12190

Selective Sonochemical post-synthesis Modification of LTA Zeolite with zinc species

PLOS ONE

Dear Dr. De Leon Ramirez,

Thank you for submitting your manuscript to PLOS ONE. After careful consideration, we feel that it has merit but does not fully meet PLOS ONE's publication criteria as it currently stands. Therefore, we invite you to submit a revised version of the manuscript that addresses the points raised during the review process.

Please submit your revised manuscript by May 25 2025 11:59PM. If you will need more time than this to complete your revisions, please reply to this message or contact the journal office at plosone@plos.org. When you're ready to submit your revision, log on to https://www.editorialmanager.com/pone/ and select the 'Submissions Needing Revision' folder to locate your manuscript file.

Please include the following items when submitting your revised manuscript:

- A rebuttal letter that responds to each point raised by the academic editor and reviewer(s). You should upload this letter as a separate file labeled 'Response to Reviewers'.
- A marked-up copy of your manuscript that highlights changes made to the original version. You should upload this as a separate file labeled 'Revised Manuscript with Track Changes'.
- An unmarked version of your revised paper without tracked changes. You should upload this as a separate file labeled 'Manuscript'.

If you would like to make changes to your financial disclosure, please include your updated statement in your cover letter. Guidelines for resubmitting your figure files are available below the reviewer comments at the end of this letter.

If applicable, we recommend that you deposit your laboratory protocols in protocols.io to enhance the reproducibility of your results. Protocols.io assigns your protocol its own identifier (DOI) so that it can be cited independently in the future. For instructions see: https://journals.plos.org/plosone/s/submission-guidelines#loc-laboratory-protocols. Additionally, PLOS ONE offers an option for publishing peer-reviewed Lab Protocol articles, which describe protocols hosted on protocols.io. Read more information on sharing protocols at https://plos.org/protocols?utm_medium=editorial-email&utm_source=authorletters&utm_campaign=protocols.

We look forward to receiving your revised manuscript.

Kind regards,

Mashallah Rezakazemi
Academic Editor
PLOS ONE

Journal Requirements:

When submitting your revision, we need you to address these additional requirements.

1. Please ensure that your manuscript meets PLOS ONE's style requirements, including those for file naming. The PLOS ONE style templates can be found at

https://journals.plos.org/plosone/s/file?id=wjVg/PLOSOne_formatting_sample_main_body.pdf and

https://journals.plos.org/plosone/s/file?id=ba62/PLOSOne_formatting_sample_title_authors_affiliations.pdf

Authors Response:
We have reviewed and corrected the format for the titles, images and tables in line with the journals style requirements.

2. Thank you for stating the following financial disclosure:
"This work was supported by the DGAPA-PAPIIT-UNAM IG101623 Project"

Please state what role the funders took in the study. If the funders had no role, please state: "The funders had no role in study design, data collection and analysis, decision to publish, or preparation of the manuscript."
If this statement is not correct you must amend it as needed.
Please include this amended Role of Funder statement in your cover letter; we will change the online submission form on your behalf.

Authors Response:
The funders role has been specified in the cover letter as suggested.

3. Please expand the acronym "UNAM" (as indicated in your financial disclosure) so that it states the name of your funders in full.
This information should be included in your cover letter; we will change the online submission form on your behalf.

Authors Response:
The acronym UNAM has been expanded stating the name of the funders in full as well as the other funders.

4. Thank you for stating the following in the Acknowledgments Section of your manuscript:

"This work was supported by the DGAPA-PAPIIT-UNAM IG101623 Project. J"

We note that you have provided funding information that is not currently declared in your Funding Statement. However, funding information should not appear in the Acknowledgments section or other areas of your manuscript. We will only publish funding information present in the Funding Statement section of the online submission form.
Please remove any funding-related text from the manuscript and let us know how you would like to update your Funding Statement. Currently, your Funding Statement reads as follows:
"This work was supported by the DGAPA-PAPIIT-UNAM IG101623 Project"

Please include your amended statements within your cover letter; we will change the online submission form on your behalf.

Authors Response:
The financial disclosure has been removed fromed from the Acknowledgments section of the manuscript and added to the cover letter for further inclusion in the Funding Statement section.

5. We notice that your supplementary figures are included in the manuscript file. Please remove them and upload them with the file type 'Supporting Information'. Please ensure that each Supporting Information file has a legend listed in the manuscript after the references list.

Authors Response:
All supplementary figures have been stated in the text as required by the style format. Equally, they have been removed from the manuscript, adding all these figures in a separate file.

[Note: HTML markup is below. Please do not edit.]

Reviewers' comments:

Reviewer's Responses to Questions

Comments to the Author

1. Is the manuscript technically sound, and do the data support the conclusions?

The manuscript must describe a technically sound piece of scientific research with data that supports the conclusions. Experiments must have been conducted rigorously, with appropriate controls, replication, and sample sizes. The conclusions must be drawn appropriately based on the data presented.

Reviewer #1: Yes

Reviewer #2: Yes

Reviewer #3: Yes

Reviewer #4: Partly

Reviewer #5: Yes

Authors Response:
We thank all reviewers for their time and comments. We are pleased that Reviewers #1, #2, #3, and #5 fully supported the manuscript. We appreciate Reviewer #4’s partial recommendation and have addressed their concerns carefully. Revisions have been made to improve clarity, methodological detail, and data interpretation. We believe the updated manuscript now meets the journal’s standards for technical rigor and scientific validity.

---

2. Has the statistical analysis been performed appropriately and rigorously?

Reviewer #1: Yes

Reviewer #2: N/A

Reviewer #3: No

Reviewer #4: N/A

Reviewer #5: Yes

Authors Response:

We appreciate the reviewer's concern regarding the statistical analysis. In response, we have revised the relevant sections to clarify the statistical methods used, including the tests applied, reporting p-values and confidence intervals where appropriate. All analyses were reviewed to ensure they were rigorous and appropriate for the data presented. These updates are highlighted in the revised manuscript.

---

3. Have the authors made all data underlying the findings in their manuscript fully available?

The PLOS Data policy requires authors to make all data underlying the findings described in their manuscript fully available without restriction, with rare exception (please refer to the Data Availability Statement in the manuscript PDF file). The data should be provided as part of the manuscript or its supporting information, or deposited to a public repository. For example, in addition to summary statistics, the data points behind means, medians and variance measures should be available. If there are restrictions on publicly sharing data—e.g. participant privacy or use of data from a third party—those must be specified.

Reviewer #1: Yes

Reviewer #2: Yes

Reviewer #3: Yes

Reviewer #4: No

Reviewer #5: Yes

Authors Response:

We thank the Reviewers for confirming that the data underlying our findings have been made fully available. As per PLOS Data Policy, all relevant data are provided within the manuscript and its Supporting Information files. We remain committed to transparency and reproducibility, and we are glad the reviewers found our data sharing practices appropriate. However, we have also stated in the "Data Availability Statement" that data can be obtained upon reasonable request.

---

4. Is the manuscript presented in an intelligible fashion and written in standard English?

PLOS ONE does not copyedit accepted manuscripts, so the language in submitted articles must be clear, correct, and unambiguous. Any typographical or grammatical errors should be corrected at revision, so please note any specific errors here.

Reviewer #1: Yes

Reviewer #2: Yes

Reviewer #3: Yes

Reviewer #4: No

Reviewer #5: Yes

Authors Response:

We thank the Reviewers for confirming the clarity and language quality of the manuscript. Nevertheless, according to all the suggestions from these, we have thoroughly revised the text to correct minor grammatical and typographical issues and to improve overall clarity. The revised manuscript has been carefully edited for standard English usage by native English speakers and Grammarly, to ensure it is clear and unambiguous throughout.

---

5. Review Comments to the Author

Please use the space provided to explain your answers to the questions above. You may also include additional comments for the author, including concerns about dual publication, research ethics, or publication ethics. (Please upload your review as an attachment if it exceeds 20,000 characters)

Reviewer #1: 1. Highlighting the roles of the species introduced into the NaA zeolite would be beneficial as to tie up all the characterization techniques that has been employed in this study. Plus the role of sonochemical treatment need to be shown that will affect the modification of properties for NaA zeolite. This can be included in the conclusion part.

Authors Response:

We have revised the Conclusion section to explicitly highlight the roles of the introduced species in modifying the NaA zeolite structure and properties, linking these effects to the results from the characterization techniques employed. Additionally, we have emphasized the impact of sonochemical treatment on enhancing dispersion, promoting ion exchange, and modifying surface characteristics. These additions help to better integrate the findings and clarify the contributions of both the introduced species and the sonochemical process.

Changes in text

The synthesized LTA zeolite in its sodium form, with a Si/Al ratio of 1:1, was successfully modified with Zn species via an ultrasonic treatment. ~~The~~This sono-assisted synthesis and support of $Zn(OH)_2$, ZnO, and ZnO_2 nanoparticles, under each specific ~~conditions, were synthesized and supported with~~condition, resulted in a high dispersion within the NaA zeolite, yielding three distinct ~~samples~~surface modifications, $Zn(OH)_2$@NaA, ZnO@NaA, and ZnO_2@NaA, respectively. The applied ultrasonic treatment did not impact the morphology of the zeolite, ~~effectively depositing the desired zinc species within the NaA structure. However, further studies are needed to increase the concentration of the deposited Zn species. It is challenging to selectively synthesize $Zn(OH)_2$ or ZnO_2 nanoparticles without obtaining the competing ZnO phase.~~nor did it compromise the crystallinity of the LTA structure. However, for sample $Zn(OH)_2$@NaA, the changes were not significant.

Con formato: Inglés (Estados Unidos), Sin Superíndice / Subíndice , Resaltar

~~The~~In contrast, in samples ZnO@NaA and ZnO_2@NaA, from the refinement data, the introduced Zn~~-modified zeolites~~ species were localized at cation exchange site I and in the α-cage, causing a cell contraction from 24.6 Å to 24.4 Å. These results correlated with the values obtained by ICP-OES and EDS, detecting Zn^{2+} in ~50% of cation exchange sites. Where the introduced ZnO or ZnO_2 species significantly enhanced the surface area (>400 $m^2\ g^{-1}$) and pore volume (>0.200 $cm^3\ g^{-1}$).

These findings suggest that the interaction of Zn species is not merely superficial but is involved in modifying the pore network of the zeolite, effectively depositing the desired zinc species within the NaA structure. This is exhibited ~~a polycrystalline structure with~~ by the distortions ~~caused by~~ of NaA when modified with the different Zn species nanoparticles. ~~Where the pore size of NaA was significantly affected, increasing both pore~~

size and surface area. Potentially enhancing the adsorptionGiven that these distortions were not detected when sonicating without Zn species precursors, the changes in the textural and optical properties of Zn-modified NaAthe zeolitic phase were attributed to the sono-assisted Zn modification.

Further studies are needed to increase the concentration of the desired Zn species in a specific local environment and to overcome the challenging selective synthesis of $Zn(OH)_2$ or ZnO_2 nanoparticles without obtaining a mixture with the competing ZnO phase. Thus, this applied ultrasonic-assisted methodology holds potential for synthesizing and supporting different types of nanoparticles on zeolites for various applications. Given that synergistic effects occur when combining zeolites with nanoparticles, which enhances the functional properties of the resulting materials. Future research is focused on optimizing the synthesis reaction by considering ultrasonic power, temperature, and time, expanding their applicability in different fieldsintroducing new functional sites that can enhance catalytic activity and potentially confer antimicrobial properties. These modifications broaden the applicability of the zeolite, making it a promising material for both biomedical and catalytic applications.

Con formato: Fuente: +Cuerpo en alfabeto complejo (Arial), Inglés (Estados Unidos), Resaltar

Con formato: Fuente: +Cuerpo en alfabeto complejo (Arial), Inglés (Estados Unidos), Resaltar

Con formato: Fuente: +Cuerpo en alfabeto complejo (Arial), Color de fuente: Texto 1, Inglés (Estados Unidos), Resaltar

Con formato: Fuente: +Cuerpo en alfabeto complejo (Arial), Color de fuente: Texto 1, Inglés (Estados Unidos), Resaltar, Ligaduras: Estándar + Contextual

2. Since this study emphasize a lot in the role of Zn species in the NaA zeolite, suggestion is to add lattice parameter data for all the exchangeable zeolites to show the significance.

Authors Response:

We appreciate the reviewer's insightful suggestion. In response, we have added the lattice parameter data and R_{wp} from the Retvield refinement for the NaA and Zn-modified zeolites to better illustrate the structural changes. This addition supports the discussion on the role of Zn species and highlights the comparative effects of the different Zn species on the NaA zeolite framework.

Changes in text

Table 3: Displacements of planes relative to NaA, where negative values indicate leftward shifts and positive values indicate rightward shifts.

Table 3. Displacements of planes relative to NaA, where negative values indicate leftward shifts and positive values indicate rightward shifts.

Plane	Peak Position (2θ)	peak position shift (Δ2θ)*		
	NaA	$Zn(OH)_2$@NaA	ZnO@NaA	ZnO_2@NaA
(200)	7.18	-0.04	0.03	0.04
(220)	10.16	-0.02	0.04	0.06
(222)	12.44	-0.02	0.09	0.11
(420)	16.09	-0.02	0.11	0.15
(620)	21.65	-0.02	0.15	0.21
(622)	23.96	-0.02	0.20	0.25
(642)	21.66	-0.04	0.15	0.19
*The values represent the peak position shift between the samples (Sample NaA).a=b=c (Å)	24.6	24.6	24.4	24.4
R_{wp}	8.5	12.8	8.1	13.1

R_{wp} = R_p- $R_{expected}$ *The values represent the peak shift difference of the samples (Δ2θ=Sample-NaA).

3. The above highlights could be use to further enhance the conclusions with regards to the final morphology of the sonochemically ion exchanges NaA zeolites.

Authors Response:

Thank you for this helpful observation. Following your suggestion, we have revised the Conclusion section to better integrate the structural data (including lattice parameters) and morphological observations. These enhancements emphasize how the nature of the Zn species and the sonochemical treatment collectively influence the final morphology and framework integrity of the NaA zeolites. This addition provides a more comprehensive summary that aligns with the study's findings and characterization results.

Reviewer #2: A comprehensive and organized investigation of the post-synthesis alteration of NaA (LTA) zeolite with zinc species utilizing a sono-assisted deposition approach is presented in the publication. The study is current and creative, providing an environmentally responsible method of customizing zeolitic materials for cutting-edge uses.

Authors Response:

Con formato: Fuente: 12 pto, Resaltar

Tabla con formato

Con formato: Fuente: 12 pto, Resaltar

Con formato: Fuente: 12 pto, Resaltar

Con formato: Fuente: 12 pto, Resaltar

Con formato: Fuente: 12 pto, Resaltar

Con formato: Fuente: 12 pto, Resaltar

Con formato: Fuente: 12 pto, Resaltar

Con formato: Fuente: 12 pto, Resaltar

Celdas insertadas

Celdas insertadas

Celdas insertadas

Celdas insertadas

Con formato: Centrado

Con formato: Fuente: 12 pto, Resaltar

We sincerely thank the reviewer for their positive and encouraging remarks regarding the novelty, environmental relevance, and organization of our study. We are pleased that the sono-assisted approach and its potential for tailoring zeolitic materials for advanced applications were well received. We have carefully considered all suggestions provided and have revised the manuscript accordingly to further improve its clarity and scientific value.

One of the key strengths of this work is the clear and systematic presentation of results. The data is well-supported with relevant references, and the findings are consistently correlated, reinforcing the reliability of the study. The discussion effectively highlights the impact of zinc species incorporation on the physicochemical properties of the modified zeolite, particularly in terms of surface charge redistribution and pore architecture.

Authors Response:

We greatly appreciate the reviewer's positive feedback on the clarity and organization of the results, as well as the consistency and reliability of the data interpretation. We are especially grateful for the recognition of our efforts to correlate the incorporation of zinc species with changes in surface charge and pore architecture. This aspect was central to our investigation, and we are pleased that it came across effectively in the discussion.

Overall, this is a well-executed and insightful piece of work that contributes to the advancement of zeolite modification strategies. The clarity of the methodology, strong correlation between results and discussion, and the practical implications of the study make this manuscript a commendable contribution to the field.

Authors Response:

We sincerely thank the reviewer for the kind and encouraging comments. We are pleased that the clarity of the methodology, the strength of the data interpretation, and the practical implications of our findings were appreciated. It is rewarding to know that our work is seen as a valuable contribution to the advancement of zeolite modification strategies.

Reviewer #3: the research topic is very interesting and could bring benefit to the scientific community. the research framework focus on the incorporation of various zinc compounds in the zeolites matrices and improving their properties. the introduction points out all the factors necessary for proper understanding of the research. the experimental part give enough information for reader to repeat synthesis and analysis of the zeolites. the results obtained are properly analyzed and discussed. the conclusion summariye all the major findings the number of figure and tables are satisfactory and give additional value to the article making it more understanable.

Authors Response:

We are grateful to the reviewer for the thoughtful and encouraging evaluation of our work. We appreciate the recognition of the relevance of the research topic and the clarity of the manuscript structure, from the introduction and experimental design to the analysis and conclusions. We are especially pleased that the reproducibility of the methods and the value of the figures and tables were acknowledged, as we aimed to make the study both rigorous and accessible to the scientific community.

what NaA means?(please describe it)

Authors Response:

Thank you for pointing this out. While "NaA" is initially defined in both the abstract and the introduction, we have revised the introduction to more clearly emphasize that NaA refers to the sodium form of zeolite A, a synthetic aluminosilicate with the LTA framework structure. This clarification ensures better understanding for all readers.

Changes in text (line 54)

This cavity diameter in zeolite A depends on the ~~cationic form, hence the commercial names 3A, 4A, and 5A for zeolites~~cations occupying the cation exchange sites, such as K^+, Na^+, or Ca^{2+} (being KA, NaA, and CaA, respectively), which allow the adsorption of molecules with kinetic diameters less than 3, 4, and 5 angstroms (Å) hence the commercial names of 3A, 4A, and 5A for zeolites, respectively [2].

what was error of determination in Tables 2?

Authors Response:

In response to this query, we have reduced the significance figures within the standard error for the given instrument and calculations. Adding to table 2 the standard deviation for the surface area and included as supporting information the pore models and parameters for the used calculations (Langmuir, t-plot and nonlocalized density functional theory).

Changes in text

Table 2. Textural properties measured from the nitrogen adsorption-desorption isotherms.

~~**Table 2: Textural properties measured from the nitrogen adsorption-desorption isotherms.**~~

Sample	S_a	S_{meso}	S_{micro}	V_P	V_{meso}	V_{micro}	D_p
	$m^2.g^{-1}$			$cm^3.g^{-1}$			nm
NaA	1.~~038~~26±0.13		~~0.019~~	0.~~016~~017	~~0.003~~		1.~~69~~09
$Zn(OH)_2$@NaA	2.75±0.~~000~~06		~~0.010~~	0.010	~~0.000~~		1.~~70~~28

Celdas eliminadas
Celdas eliminadas
Con formato: Fuente: 12 pto
Con formato: Fuente: 12 pto
Con formato: Fuente: 12 pto
Con formato: Fuente: 12 pto
Con formato: Fuente: 12 pto
Con formato: Fuente: 12 pto
Celdas eliminadas
Celdas eliminadas
Con formato: Fuente: 12 pto
Con formato: Fuente: 12 pto, Ligaduras: Estándar + Contextual
Con formato: Fuente: 12 pto
Con formato: Fuente: Calibri, 12 pto, Color de fuente: Negro, Ligaduras: Ninguna
Con formato: Fuente: 12 pto
Con formato: Fuente: 12 pto
Con formato: Fuente: 12 pto
Con formato: Fuente: Calibri, 12 pto, Color de fuente: Negro
Con formato: Fuente: 12 pto
Con formato: Fuente: 12 pto
Con formato: Fuente: 12 pto
Con formato: Fuente: 12 pto
Con formato: Fuente: 12 pto
Con formato: Fuente: 12 pto
Con formato: Fuente: 12 pto
Con formato: Fuente: 12 pto
Con formato: Fuente: 12 pto

ZnO@NaA	403437.88±3.48	403.46079.40	0.000358.48	0.015267	0.150157	0.000110	10.91
ZnO_2@NaA	581.42±0.86	240.80011.31	340.610570.11	0.224	0.380025	0.180199	0.20091

All the values were rounded to the minimum significant digits for convenience and simplification.S_a=surface area (Langmuir) , S_{meso}=mespore surface area(T plot), S_{micro}= micropore surface area(T plot), V_p= pore volume(DFT), V_{meso}= mesopore volume(T plot), V_{micro}= micropore volume(T plot), D_p= pore diameter

3.5 All the values were rounded to the minimum significant digits for convenience and simplification. S_a=surface area (Langmuir) , S_{meso}=mespore surface area(*t-plot*), S_{micro}= micropore surface area(*t-plot*), V_p= pore volume(NLDFT), V_{meso}= mesopore volume(*t-plot*), V_{micro}= micropore volume(*t-plot*), D_p= pore diameter (NLDFT)

what were the major benefits of the Zn incorporation comparing to the LTA?

Authors Response:

In response to this comment, the information regarding surface are and active sites for catalytic and biomedical applications has been integrated into the revised discussion and conclusion sections for clarity.

Reviewer #4: Peer Review PONE-D-25-12190

The manuscript entitled “Selective Sonochemical Post-Synthesis Modification of LTA Zeolite with Zinc Species” employs a sonochemical approach to modify LTA zeolite by depositing zinc-based nanoparticles.

In my opinion, this work is interesting, but I suggest major revisions before further consideration.

GENERAL OBSERVATIONS: The manuscript presents an interesting study; however, it contains numerous typos, grammatical errors, and lacks clarity regarding the experimental conditions. I strongly recommend a thorough review of the text.

Author Response:

We thank the reviewer for the valuable feedback. In response, we have carefully revised the entire manuscript to correct typographical and grammatical errors and to improve the overall clarity and readability. Additionally, we have clarified and expanded the descriptions of the experimental conditions to ensure they are detailed enough for reproducibility. We hope these revisions address the reviewer’s concerns and enhance the quality of the manuscript.

Additionally, it remains unclear what type of interaction exists between the zeolite and zinc species (ion exchange, surface deposition, deposition within cavities, etc.). Furthermore, the practical applicability of this composite material is not well elucidated.

Authors Response:

Celdas eliminadas
Celdas eliminadas
Con formato: Fuente: 12 pto
Con formato: Fuente: 12 pto
Con formato: Fuente: 12 pto
Con formato: Fuente: 12 pto
Con formato: Fuente: 12 pto
Con formato: Fuente: 12 pto
Con formato: Fuente: 12 pto
Con formato: Fuente: 12 pto
Con formato: Fuente: 12 pto
Con formato: Fuente: 12 pto
Con formato: Fuente: 12 pto
Celdas eliminadas
Celdas eliminadas
Con formato: Fuente: 12 pto
Con formato: Fuente: 12 pto
Con formato: Fuente: +Cuerpo (Aptos), 12 pto, Color de fuente: Automático, Ligaduras: Estándar + Contextual
Con formato: Fuente: 12 pto
Con formato: Fuente: Calibri, 12 pto, Color de fuente: Negro, Ligaduras: Ninguna
Con formato: Fuente: 12 pto
Con formato: Fuente: +Cuerpo (Aptos), 12 pto, Color de fuente: Automático, Ligaduras: Estándar + Contextual
Con formato: Fuente: +Cuerpo (Aptos), 12 pto, Color de fuente: Automático, Ligaduras: Estándar + Contextual
Con formato: Fuente: 12 pto
Con formato: Fuente: 12 pto
Con formato: Fuente: 12 pto

We agree and in pursuit to clearly manifest these interactions, we have revised the discussion section to better clarify the nature of the interaction between the Zn species and the zeolite matrix. Based on our characterization results, we discuss the contributions of both ion exchange and surface/cavity deposition, depending on the Zn precursor used and the conditions of sonochemical treatment. Additionally, we have expanded the conclusion section to more clearly address the potential practical applications of the modified zeolites, particularly in areas such as catalysis, environmental remediation, or antimicrobial materials, supported by relevant literature.

Changes in text

The synthesized LTA zeolite in its sodium form, with a Si/Al ratio of 1:1, was successfully modified with Zn species via an ultrasonic treatment. ~~The~~This sono-assisted synthesis and support of $Zn(OH)_2$, ZnO, and ZnO_2 nanoparticles, under each specific ~~conditions, were synthesized and supported with~~condition, resulted in a high dispersion within the NaA zeolite, yielding three distinct ~~samples~~surface modifications, $Zn(OH)_2$@NaA, ZnO@NaA, and ZnO_2@NaA, respectively. The applied ultrasonic treatment did not impact the morphology of the zeolite, ~~effectively depositing the desired zinc species within the NaA structure. However, further studies are needed to increase the concentration of the deposited Zn species. It is challenging to selectively synthesize $Zn(OH)_2$ or ZnO_2 nanoparticles without obtaining the competing ZnO phase.~~nor did it compromise the crystallinity of the LTA structure. However, for sample $Zn(OH)_2$@NaA, the changes were not significant.

~~The~~In contrast, in samples ZnO@NaA and ZnO_2@NaA, from the refinement data, the introduced Zn~~-modified zeolites~~ species were localized at cation exchange site I and in the α-cage, causing a cell contraction from 24.6 Å to 24.4 Å. These results correlated with the values obtained by ICP-OES and EDS, detecting Zn^{2+} in ~50% of cation exchange sites. Where the introduced ZnO or ZnO_2 species significantly enhanced the surface area (>400 $m^2\ g^{-1}$) and pore volume (>0.200 $cm^3\ g^{-1}$).

Con formato: Inglés (Estados Unidos), Sin Superíndice / Subíndice , Resaltar

These findings suggest that the interaction of Zn species is not merely superficial but is involved in modifying the pore network of the zeolite, effectively depositing the desired zinc species within the NaA structure. This is exhibited ~~a polycrystalline structure with~~ by the distortions ~~caused by~~ of NaA when modified with the different Zn species nanoparticles. ~~Where the pore size of NaA was significantly affected, increasing both pore size and surface area. Potentially enhancing the adsorption~~Given that these distortions were not detected when sonicating without Zn species precursors, the changes in the textural and optical properties of ~~Zn-modified NaA~~the zeolitic phase were attributed to the sono-assisted Zn modification.

Further studies are needed to increase the concentration of the desired Zn species in a specific local environment and to overcome the challenging selective synthesis of $Zn(OH)_2$ or ZnO_2 nanoparticles without obtaining a mixture with the competing ZnO phase. Thus, this applied ultrasonic-assisted methodology holds potential for synthesizing and supporting different types of nanoparticles on zeolites for various applications. Given that synergistic effects occur when combining zeolites with nanoparticles, which enhances the functional properties of the resulting materials. Future research is focused on optimizing the synthesis reaction by considering ultrasonic power, temperature, and time, ~~expanding their applicability in different fields~~introducing new functional sites that can enhance catalytic activity and potentially confer antimicrobial properties. These modifications broaden the applicability of the zeolite, making it a promising material for both biomedical and catalytic applications.

Abstract

• The sentence: “In this study, we employed an eco-friendly post-synthesis modification of a NaA zeolite with zinc species: ZnO, ZnO_2, and $Zn(OH)_2$ by a sono-assisted deposition method. This

Con formato: Fuente: +Cuerpo en alfabeto complejo (Arial), Inglés (Estados Unidos), Resaltar

Con formato: Fuente: +Cuerpo en alfabeto complejo (Arial), Inglés (Estados Unidos), Resaltar

Con formato: Fuente: +Cuerpo en alfabeto complejo (Arial), Color de fuente: Texto 1, Inglés (Estados Unidos), Resaltar

Con formato: Fuente: +Cuerpo en alfabeto complejo (Arial), Color de fuente: Texto 1, Inglés (Estados Unidos), Resaltar, Ligaduras: Estándar + Contextual

approach significantly reduced synthesis time.”
o The meaning of "synthesis time" is unclear; please clarify what is being referred to.

Authors Response:

The term “synthesis time” refers specifically to the deposition time of the zinc species onto the NaA zeolite. In the sono-assisted method employed, ultrasonic irradiation promotes rapid nucleation and interaction between the precursor and the zeolite surface, thereby significantly reducing the overall duration required for the deposition process when compared to conventional methods. We have clarified this point in the revised manuscript and add the process time.

Changes in text

Abstract

LTA zeolite is known for its straightforward synthesis and well-defined structure, composed of α and β cages. This structure offers a versatile platform for hosting molecules of varying sizes. In the sodic form (NaA~~)~~), the Na^+ ions are located ~~in the~~within these cages' cation exchange sites~~ within these cages~~. When substituted with zinc species, a precise tuning of pore size and properties can emerge. In this study, we employed an eco-friendly post-synthesis modification of a NaA zeolite with zinc species: ZnO, ZnO_2, and $Zn(OH)_2$ by a sono-assisted deposition method. This approach ~~significantly reduced synthesis~~allowed a short deposition time~~,~~ (30 min), providing control over the desired Zn species ~~desired to be~~ incorporated into NaA ~~(LTA)~~ zeolite. Equally, the facile selective integration of zinc species in the form of supported nanoparticles and Zn^{2+} in cation exchange sites modified the physicochemical properties of the material, including unique surface charge redistribution and pore architectures. These results highlight the potential of sono-assisted deposition as an alternative strategy for engineering advanced zeolitic materials, opening new avenues for innovative applications in catalysis, adsorption, and beyond.

• Provide additional information on the interaction between LTA zeolite and Zn species.

Authors Response:

We appreciate the reviewer’s interest in this point. The interaction between the Zn species and the LTA zeolite occurs primarily through two mechanisms: (i) incorporation of Zn^{2+} ions at cation exchange sites within the zeolite framework, and (ii) deposition of Zn-based nanoparticles on the external surface and partially within the pore system. These interactions were inferred based on the combined results of EDX, XRD, and spectroscopic analyses. Additional clarification has been added

to the revised manuscript to emphasize these points.the interaction of the Zn species with the zeolite was in the form of nanoparticles and Zn^{2+} in cation exchange sites.

Changes in text

The changes in the diffractograms associated ~~to~~with the interactions between the nanoparticles and the zeolitic matrix~~,~~ consist of intensities of the crystallographic planes of the samples (~~Figure~~Fig 4B). ~~Where a~~A decrease in intensity is observed in the (220), suggesting the presence of Zn ions at exchange site I in the NaA zeolite (~~Figure~~Fig 4C). On the other hand, the patterns show an increase in the reflection of the (400), indicating the possible incorporation of Zn within the α-cage of the zeolite (~~Figure~~Fig 4D). Confirming that these changes in the reflection of specific planes are attributed to the interaction of foreign species (ZnO, ZnO_2, or $Zn(OH)_2$) with the zeolite structure ~~[49]~~[59].

In general, the detected interaction by XRD showed that Zn species interact with the zeolite crystal lattice, evidenced by a significant decrease ($p < 0.05$) in the intensity of plane (220), an increase in plane (400), and angular shifts in diffractograms. These shifts show a slight reduction in the unit cell parameters, with lattice constants decreasing from 24.6 Å in pristine NaA and $Zn(OH)_2$@NaA to 24.4 Å in ZnO@NaA and ZnO_2@NaA, indicating lattice contraction likely due to partial incorporation of Zn species.

Introduction

• Lines 81-87: Review these sentences for clarity and grammatical correctness.

Authors Response:

We appreciate and agree with the reviewers observation. The sentences in lines 81–87 have been carefully revised to improve clarity and ensure grammatical accuracy. The updated text now reads more fluently and better conveys the intended meaning.The sentences were rewritten to ensure clarity and grammatical correctness.

Changes in text

Ultrasound can influence various stages of the synthesis process of zeolitic materials: before, during, and after crystallization. The application of sonochemical

methods has gained relevance in the fields of crystallization, due to its ability to enhance and accelerate crystal formation by reducing the induction period. These improvements are essential for achieved through acoustic cavitation, where ultrasonic waves induce the collapse of bubbles that generate high-energy microenvironments, releasing free radicals that promote controlled nucleation and accelerate crystal growth. This approach, known as sonocrystallization, allows for a reduction in synthesis time, a decrease in average crystal size, improved particle size distribution, and better control over material morphology. Furthermore, the simplicity and efficiency of this method make it a promising alternative to conventional hydrothermal methods for obtaining zeolitic materials [28].

Con formato: Color de fuente: Negro, Inglés (Estados Unidos), Resaltar

Experimental

• Line 94: Specify the precursor used.

Authors Response:

Thank you for pointing this out. The specific zinc precursor used has now been clearly stated in the methodology section to improve transparency and reproducibility.

Changes in text

LTA zeolite was synthesized using a hydrothermal method based on the procedure proposed by R. W. Thompson and K. C. Franklin [26][36], with slight precursor modifications. Crystallization: NaOH (Catalog No. 221465), Al(OH)$_4$Na (Catalog No. 13404) and $Na_2O(SiO_2)x{\cdot}xH_2O$ (Catalog No.338443) were supplied by Sigma Aldrich. Initially, sodium hydroxide (NaOH, ≥98%, Sigma-Aldrich) was performed at 99 °C for dissolved in 4 hoursL of deionized water under static conditions, followed by a decantation washing stepgentle stirring until a stable pH of the supernatant reached a value of 8.4. Finally, the material was dried in an oven at 80°C for 24 hours, and thefully dissolved. The resulting zeolite powder, in the sodium form, was designated as NaAsolution was equally divided into two 2 L portions and transferred into polypropylene bottles.

2.1.2 Deposition of zinc species

NaA was used for the deposition of zinc species by the sonoassisted method, with the required adaptations adjusted to each desired zinc species, such as concentration and pH, as obtained from the speciation diagrams (Figure A1). The desired zinc species, zinc hydroxide $(ZnOH)_2$, zinc oxide (ZnO) and zinc peroxide (ZnO_2) species were deposited as follows.

For each zinc speciesOne portion of sodium aluminate ($NaAlO_2$, Sigma-Aldrich), sodium aluminate ($NaAlO_2$, Sigma-Aldrich) was added and mixed in a sealed container until a clear solution was obtained. In the second portion, commercial sodium silicate solution was added in sufficient amounts to maintain an appropriate Si/Al molar ratio. The mixture was stirred until homogeneous. The sodium silicate solution was quickly poured into the aluminate solution, forming a viscous gel. The container was tightly sealed and manually shaken until a uniform gel was obtained. The gel was transferred to a polypropylene bottle and subjected to hydrothermal crystallization treatment at 99 ± 1 °C for 4 hours under static conditions.

After crystallization, the suspensions were allowed to cool to room temperature (<30 °C), and the solid product was recovered by decantation. The solids were washed with excess deionized water until the supernatant reached a pH<9. Finally, the material was dried by separating on different filter papers and placed on a watch glass at 80°C for 24 hours. The final yield was approximately 500 g of dry material, and the resulting zeolite powder in the sodium form was designated as NaA.

Line 100: Correct “sonoassisted” to “sono-assisted” if necessary.

Author's Response:

Following the manuscript revision, the term “sonoassisted” has been corrected to “sono-assited” at line 100. Equally, we have carefully reviewed the entire text to ensure consistent and correct use of hyphenation, including “sono-assisted,” and to address any remaining grammatical issues.

• Properly comment on Figure A1 before describing modification methods.

Authors Response:

We appreciate the suggestion. The section discussing S1 Fig (preceding Figure A1) has been revised to include a more thorough explanation, providing better context before introducing the modification methods.

Changes in text

The speciation diagrams were modeled using the Hydra/Medusa software, illustrating the fraction of zinc species at different pH values and concentrations (S1 Fig). Based on these diagrams, the concentration and pH conditions were carefully adjusted to favor the desired zinc species' selective deposition by promoting a specific species' predominance under controlled conditions. It was shown that $Zn(OH)_2$ formation is favored at low zinc concentrations. In contrast, ZnO becomes the predominant species at higher concentrations and under alkaline conditions. Hence, this specific predominance promoted the desired zinc species' selective deposition.

• Line 116: “Adjusting the final pH (according to each Zn species) and volume to 50 mL” — clarify this procedure.

Authors Response:

Thank you for your observation. We have clarified the procedure in the revised manuscript to specify how the final pH was adjusted based on the zinc precursor used and how the final solution volume was brought to 50 mL. This ensures the methodology is more transparent and reproducible.

Changes in text

In brief, NaA was suspended in water, and by a sono-assisted method, $Zn(OH)_2$, ZnO, or ZnO_2 were synthesized and deposited on the zeolitic surface. Initially, 1 g of NaA was ~~initially~~ dispersed in 25 mL of water using ~~ultrasonication for 5 min.~~ultrasonic

irradiation for 5 min and preheated to 65 ºC (step done in triplicate, for each Zn species). For the precipitation of $Zn(OH)_2$, 10 mL of 0.05 mM solution of zinc acetate ($Zn(CH_3COOH)_2$) was added dropwise to the 25 mL zeolitic suspension, adjusting theto a final pH value toof 12, with concentrated NH_4OH [27][37]. For the deposition of ZnO species, 25 mL of 0.2 mM aqueous solution of $Zn(CH_3COOH)_2$ was prepared and added to a 25 mL zeolitic suspension, carefully adjusting the final pH value to 8, employing acetic acid or NH_4OH when necessary [25][33]. For ZnO_2, 1g of zinc acetate was dispersed in 20 mL of distilled water, preheated to 65 ºC, and mixed under ultrasound for 2 min with 5 mL of 3% hydrogen peroxide [28][38]. Once all the zinc precursor solutions were prepared, each was , and added dropwise to a separate 25 mL NaA suspension preheated to 65 ºC, adjusting the . The final pH (accordingfor all the prepared suspensions was adjusted during the dropwise addition of the Zn precursor solutions with concentrated HCl or NH_4OH. Once the desired pH was reached, H_2O was added to each Zn species) and volume tothe 50 mL mark and allowed to react under ultrasonic irradiation for 30 min at 65 ºC. The well-formed precipitates were filtered under vacuum through a 0.45 µm membrane and washed with excess distilled water until a neutral value (pH=7) was achieved. The resulting products were then dried at 35 ºC for 24 hours and labeled $Zn(OH)_2$@NaA, ZnO@NaA, and ZnO_2@NaA[39].

Con formato: Color de fuente: Negro, Inglés (Estados Unidos), Resaltar

Con formato: Color de fuente: Negro, Inglés (Estados Unidos), Resaltar

• The passage: “To confirm the effect of the preparation conditions on the zeolite structure, blank samples (excluding the addition of Zn^{2+} precursor solution) were prepared at the deposition solutions (pH, ultrasound duration, and temperature) and studied by X-ray diffraction analysis. Given that no difference was observed, these samples were excluded from the study, presenting the data as an appendix (Figure A2).”

o This statement is unclear; please rephrase for better readability.

Authors Response:

We appreciate the reviewer's comment. The passage has been rewritten, according to all reviewers for improved clarity. This revised version ensures the procedure and rationale are easier to follow. Making all the methodology section more clear, resolving any ambiguity.

Changes in text

To confirmevaluate the effect of the preparationsonication conditions on the zeolite structure, blank samples (excluding the addition of Zn^{2+} precursor solution) were prepareda NaA suspension was sonicated at the deposition solutionsconditions (pH, ultrasound duration, and temperature) and studied), with no addition of Zn^{2+} precursor (Blank samples). These blank samples, (OH)@NaA and (O_2)@NaA, were analyzed by X-ray diffraction analysis. Given, and given that no difference was observed, these samples were excluded from the study, presenting the . This data is presented as an appendix (Figure A2support information (S2 Fig).

• The section on microscopy techniques: "Transmission electron microscopy (TEM) measurements (Hitachi H7500, Hitachi Ltd., Tokyo, Japan) at 80 kV. Sample morphology was obtained by scanning electron microscopy (SEM) using a JEOL JIB-4500 (MA, USA). The images were processed in Image J imaging software, and a second set of samples was analyzed on a LEICA Stereoscan 440 Scanning Electron Microscope. The samples were pretreated with a gold sputtering coating. The energy dispersive X-ray spectroscopy (EDX) coupled to the microscope used for SEM was also recorded."
o This section requires revision; specify the EDX instrument used and clarify why two different SEM instruments were employed for two different sets of samples. Which samples were analyzed by each method? The same applies to ICP-OES.

Authors Response:

We acknowledge and have corrected our mistake. The original text mistakenly referred to two different SEM instruments. We confirm that only one SEM instrument—the JEOL JIB-4500—was used in this study. We have also clarified the details of the EDX system coupled to this microscope. Additionally, the information on the ICP-OES methodology has also been specified, describing important details.

Changes in text

Transmission electron microscopy (TEM) measurements (Hitachi H7500, Hitachi Ltd., The Tokyo, Japan) at 80 kV. Sample morphology of all samples was obtained byfrom

scanning electron microscopy (SEM) using a measurements (JEOL JIB-4500 (equipment, MA, USA). The images were processed in Image J imaging software, and a second set of samples was analyzed on LEICA Stereoscan 440 Scanning Electron Microscope. The samples were pre-treated with a gold sputtering coating. The energy); the same equipment was used to obtain the chemical analysis data by X-ray dispersive X-rayenergy spectroscopy (EDX). These data were compared with transmission electron microscopy (TEM, Hitachi H7500, Hitachi Ltd., Tokyo, Japan) at 80 kV.) coupled to Calculating the microscope used for SEM was also recordedparticle size distribution by treating the micrographs using the ImageJ software.

• "A quantity between 25-50 mg was dissolved..." — clarify what was dissolved.

Authors Response:

We appreciate the reviewer's attention to this detail. The sentence has been revised for clarity and now specifies that each sample was independently dissolved, obtaining the digested zeolitic material.

Changes in text

The chemical composition of the samples was evaluated by inductively coupled plasma-optical emission spectroscopy (ICP-OES) using a Vista-MPX CCD (CO, USA) simultaneously with ICP-OES (Varian) (CO, USA). A quantity between 25-50 mg of each sample (NaA, $Zn(OH)_2$@NaA, ZnO@NaA, or ZnO_2@NaA) was dissolved by adding 2 mL of HNO_3 and 1 mL of concentrated HF and left for 24 h at 40 ºC. Subsequently, 40 mL of 2% H_3BO_3 was added and replaced for 5 h at 40 ºC. Before the measurements, all samples were diluted by a factor of 25. The atomic weight percentages were obtained by calculating the number of moles of each element divided by the total moles of all elements in the formula.

• "The samples were degassed at 50°C for 60 minutes, followed by a heating ramp with a rate of 5°C min^{-1} until reaching 250°C, maintaining this temperature for 360 minutes before the analysis."
o Explain why this thermal treatment was performed.

Authors Response:

In response to this is helpful point, the physisorption methodology has been rewritten, explaining relevant details. We ensured that the readers can understand that the thermal treatment was carried out to ensure complete removal of physically adsorbed moisture and volatile contaminants from the zeolite surface and pores prior to nitrogen adsorption analysis. Explaining that the proper degassing is essential for obtaining accurate surface area and porosity measurements.

Changes in text

N_2 adsorption-desorption isotherm measurements were used to determine the textural properties ~~were measured on~~ , surface area (S_a), *pore volume* (V_p), and pore diameter (D_P) of the studied materials at −196 °C with P/P_0 values of 0–0.99 in a Quantachrome Instruments autosorb iQ~~, using nitrogen as an adsorbent, and the data were processed with the Quantachrome ASiQwin software. The samples were~~ device. The studied materials were degassed at 50°C for 60 minutes, followed by a heating ramp with a rate of 5°C min^{-1} until reaching 250°C, maintaining this temperature for 360 minutes ~~before the analysis.~~. The data was processed with the Quantachrome ASiQwin software using the Langmuir equation to calculate the specific surface areas (S_a) and NLDFT for the cumulative pore volume (V_p) from the saturation point (P/P_0 ~ 0.7). For micropore area and volume (S_{micro} and V_{micro}), a *t-plot* was used along with the NLDFT data to model the micropore size distribution and calculate the mean pore diameter (D_P). The mesopore area ($S_{meso} = S_a - S_{micro}$) and volume ($V_{meso} = V_p - V_{micro}$) were obtained from the differences between the total and micropore values. The obtained models and standard deviations are presented as supporting information (S1 Table).

• "The study was conducted from 30°C to 800°C, with a heating ramp of 20°C min^{-1} in an O_2 atmosphere."
o Specify whether the atmosphere was pure O_2 or a mixture (e.g., N_2/O_2).

Authors Response:

We give thanks for pointing this out information, in the revised manuscript we have clarified that the atmosphere used during the thermal analysis was pure O_2. This detail has been added to the revised version to avoid any ambiguity. The themogravimetry analysis has been specified in detail

Changes in text

The thermal stability of the prepared zeolites was evaluated using (TGA analysis),, performed with a TA instrument, model Q600. The study was conducted in an O_2 atmosphere from 30 °C to 800 °C, with a heating ramp withat a rate of 20 °C min^{-1} in an O_2 atmosphere. To determine the zeta potential (ζ), the samples were resuspended in water with the help of an ultrasonic bath at room temperature (>5 min) until a stable suspension was reached. The ζ measurements were then performed in Zetasizer Nano ZS (from Malvern Instruments) by averaging three automatic readings at 25 ºC, with no pause between measurements. Diffuse reflectance spectroscopy (DRS) measurements in the ultraviolet-visible (UV-Vis) range were performed using a Cary 5000 UV-Vis NIR spectrophotometer (Richmond, CA, USA) in a wavelength range of 200–1200 nm at room temperature. The band gap (E_g) was calculated from thesethe obtained spectra by applying the Kubelka-Munk function [29][40].

Con formato: Color de fuente: Negro, Inglés (Estados Unidos), Ligaduras: Ninguna

• Provide additional details regarding the experimental conditions for FTIR and Raman spectroscopy.

Authors Response:

In agreement, more specific experimental conditions have been specified to improve reproducibility. In the revised manuscript, we have included the key parameters for both techniques. Such as the amount of sample used, number of scans and average readings for FTIR and nominal power of the laser, magnification of the used lens and the integration time for the acquisition of Raman spectra.

Changes in text

To study the binding vibrations of the Zn species and the zeolite, Fourier transform infrared spectroscopy (FT-IR) and Raman spectroscopy were employed. The spectra were obtained by a Nicolet IS10 Thermo Scientific FT-IR equipment (Waltham, MA, USA), with the ATR attachment loading approximately 100 mg of powder sample. The method used was a sweep of 500 cm^{-1} to 4000 cm^{-1} (100 scans were performed per reading, and 3 readings were carried out). Raman spectra were collected using a LabRAM HR 800 confocal micro-Raman spectrometer (Horiba Jobin-Yvon, HORIBA Instruments Inc., Piscataway, NJ, USA), equipped with an OLYMPUS BX41 microscope. The samples were placed on the microscope stage and examined at room temperature. A 633 nm He-Ne laser was employed as the excitation source, with a nominal output power of 17 mW, attenuated using a neutral density filter (D3, I/1000) to prevent thermal damage to the sample. Spectra were acquired from at least three points on each sample to ensure reproducibility. A 100× objective lens focused the laser beam on the material's surface. The integration time was 8 seconds, and the spectra were collected in the 100-1500 cm^{-1} range.

Con formato: Inglés (Estados Unidos), Diseño: Claro, Resaltar, Ligaduras: Ninguna

• Figure 3: The authors present N_2 sorption/desorption isotherms, but this method is not described in the experimental section. A proper description should be included.

Authors Response:

We appreciate the reviewer's observation. In response, we have added a detailed description of the N_2 adsorption-desorption methodology in the corresponding section of the revised manuscript. This modification ensures that the characterization method for the isotherms in Figure 3 is properly documented in the experimental section.

Changes in text

N_2 adsorption-desorption isotherm measurements were used to determine the textural properties ~~were measured on~~ , surface area (*S_a*), *pore volume* (*V_p*), and pore diameter (*D_P*) of the studied materials at −196 °C with P/P_0 values of 0–0.99 in a

Quantachrome Instruments autosorb iQ, using nitrogen as an adsorbent, and the data were processed with the Quantachrome ASiQwin software. The samples were device. The studied materials were degassed at 50°C for 60 minutes, followed by a heating ramp with a rate of 5°C min^{-1} until reaching 250°C, maintaining this temperature for 360 minutes before the analysis.. The data was processed with the Quantachrome ASiQwin software using the Langmuir equation to calculate the specific surface areas (S_a) and NLDFT for the cumulative pore volume (V_p) from the saturation point ($P/P_0 \sim 0.7$). For micropore area and volume (S_{micro} and V_{micro}), a *t-plot* was used along with the NLDFT data to model the micropore size distribution and calculate the mean pore diameter (D_P). The mesopore area ($S_{meso} = S_a - S_{micro}$) and volume ($V_{meso} = V_p - V_{micro}$) were obtained from the differences between the total and micropore values. The obtained models and standard deviations are presented as supporting information (S1 Table).

Results and Discussion

• The term "thermal stability" is unclear. The samples appear to be thermally unstable, as they exhibit significant weight loss at low temperatures. Thermal stability (or oxidative stability in this case) should be defined based on onset temperature from DTG curves.

Author response:

We thank the reviewer for pointing out the ambiguity in our use of the term "thermal stability." In the revised manuscript, we clarified that while the samples do exhibit initial weight loss due to water desorption, they do not undergo significant structural degradation up to 800 °C. Accordingly, we have refined the definition of thermal stability to refer to the absence of framework decomposition, as inferred from the DTG onset temperature.

Changes in text

The comparison of the thermogravimetric analysis's profiles (Figure 6BFig 6A) to zeolite NaA and the ultrasonic-treated samples demonstrates the high thermal stability (indicates that all samples maintain structural integrity up to 800 °C) for all samples., despite initial weight loss due to water desorption. Type NaA zeolites exhibit two main

regions of ~~water~~mass loss, one ~~at about~~near, 130 ~~degrees Celsius~~°C and ~~the other at about~~another around, 200 °C, which ~~may correspond to~~ are commonly associated with the loss of physisorbed water molecules ~~adsorbed on~~from the surface and internal pores of the ~~zeolites~~zeolite framework[90]. Although these materials exhibit early weight loss, no significant decomposition or ~~within the pores [80]~~framework collapse is observed up to 800 °C, suggesting high thermal robustness. For clarity, thermal stability in this context refers to the absence of structural degradation, as determined by the decomposition onset temperature in DTG curves.

Con formato: Inglés (Estados Unidos), Resaltar, Ligaduras: Estándar + Contextual

Con formato: Inglés (Estados Unidos), Resaltar, Ligaduras: Estándar + Contextual

• Sample ZnO_2@NaA exhibits a weight gain at higher temperatures, which may be due to nitrogen adsorption from the furnace atmosphere, as also observed in isotherm measurements.

Authors Response:

The observed weight gain in the ZnO_2@NaA sample at elevated temperatures has been attributed to oxidative processes, rather than nitrogen adsorption, since the TGA experiments were conducted in a pure O_2 atmosphere. This clarification has been added to the revised manuscript in the Results and Discussion section to provide a more accurate interpretation of the data.

Changes in text

This can be attributed to the particle size within the structure, as it may block the release of water from the zeolite channel system ~~[82,83]~~[92,93]~~. The~~. All materials exhibit similar mass loss ~~behavior, decreasing from 90 °C to 450 °C, attributed to~~ profiles associated with the ~~abovementioned~~aforementioned phenomena~~. It was observed that the~~ between 90 °C and 450 °C. Interestingly, a slight increase in final mass was ~~higher in~~ observed in the nanoparticle-containing samples, particularly ZnO_2@NaA. This behavior may be attributed to oxidative processes increasing the ~~nanoparticles, and this effect has been previously reported [84]~~observed final weight [94].

Con formato: Color de fuente: Color personalizado(RGB(46,46,46)), Inglés (Estados Unidos), Resaltar

• Figure 6: It is recommended to invert the order of A and B to align with the discussion in the text. Overall, the manuscript requires substantial revision for clarity, grammar, and experimental detail. The authors should ensure that all methods are thoroughly described and justified, particularly regarding sample preparation and characterization techniques.

Authors Response:

We thank for this constructive suggestion, and have matched the text to the order of panels A and B in figure 6 to align better with the flow of the discussion in the text.

Additionally, we have thoroughly revised the manuscript for clarity, grammar, and consistency. Particular attention was given to the Materials and Methods section, where we expanded the descriptions of sample preparation and characterization procedures to ensure full transparency and reproducibility.

Changes in text

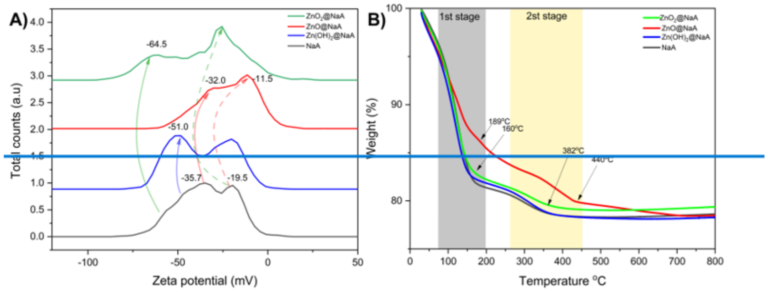

Figure

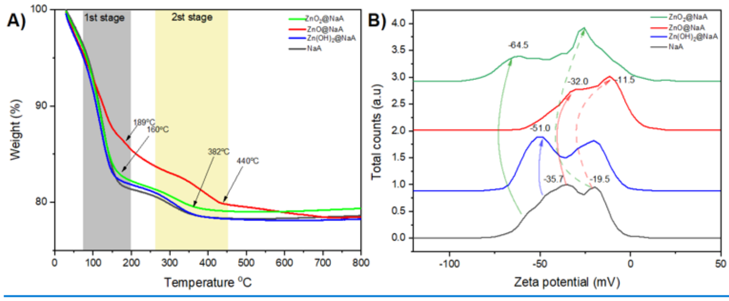

Fig **6**:. **Zeta potential (ζ)** measurements. A) and thermogravimetric analysis (TGA) of NaA B) modified with $Zn(OH)_2$ (blue line), ZnO (red line)), or ZnO_2 (green line).

Reviewer #5: Dear Editor,

I have carefully read the manuscript PONE-D-25-12190, entitled "Selective Sonochemical Post-Synthesis Modification of LTA Zeolite with Zinc Species", by J. I. De León-Ramírez et al., submitted to PLOS ONE.

Overall, I found this manuscript interesting, and I believe it merits publication in PLOS ONE after revision.

- First, the entire document should be reviewed by a native English speaker. The structure of some paragraphs is clearly inadequate but can be easily corrected. This would improve the readability and coherence of the text.

Author response:

We appreciate the reviewer's suggestion regarding language and clarity. A native English speaker has thoroughly revised the manuscript to improve grammar, structure, and overall readability.

- The authors should improve the flow of the text around line 67. There is a noticeable gap that disrupts the reading at this point.

Author response:

We thank the reviewer for pointing out the issue with the flow of the text around line 67. We have revised this section to improve coherence and ensure a smoother transition between ideas, enhancing the overall readability of the introduction.

Changes in text

LTA zeolite's exceptional ion-exchange properties and high surface area make it a widely used material in applications such as water purification, gas adsorption, and catalysis[5–10]. A key focus of recent studies is the functionalization of LTA by incorporating nanoparticles to enhance its properties. Such modifications often exploit the zeolite's considerable nanoporosity, of zeolite, which for LTA is approximately 48%%, free space in the case of LTA,that serves to host nanoparticles while preventingand prevent their agglomeration [11–13].

Zinc-based species Researchers have explored Zn-based compounds such as ZnO [14–17], $Zn(OH)_2$ [18,19], and ZnO_2 [20–22] to develop advanced materials for various applications. These zinc-based nanoparticles have garnered significant attention among these nanoparticles for their catalytic, photocatalytic, and oligodynamic properties. Zinc's interactionsThe interaction of Zinc with biological systems further underscoreunderscores its relevance. The ion exchange of Zn^{2+} cations in LTAConsidering that ZnO is straightforward. Researchers have explored the incorporation of Zn-based compounds such as ZnO [14–17], $Zn(OH)_2$ [18,19], and ZnO_2 [20–22] intomain compound of interest in the literature that is often stabilized and most studied within zeolite matrices to develop advanced materials for various applications.

Con formato: Fuente: +Cuerpo en alfabeto complejo (Arial), Inglés (Estados Unidos), Resaltar

Con formato: Fuente: +Cuerpo en alfabeto complejo (Arial), Inglés (Estados Unidos), Resaltar

Con formato: Fuente: +Cuerpo en alfabeto complejo (Arial), Inglés (Estados Unidos), Resaltar

Con formato: Fuente: +Cuerpo en alfabeto complejo (Arial), Inglés (Estados Unidos), Resaltar

Con formato: Fuente: +Cuerpo en alfabeto complejo (Arial), Inglés (Estados Unidos), Resaltar

Con formato: Fuente: +Cuerpo en alfabeto complejo (Arial), Inglés (Estados Unidos), Resaltar

Con formato: Fuente: +Cuerpo en alfabeto complejo (Arial), Inglés (Estados Unidos), Resaltar

Post (ZnO@LTA) [23–27], we propose an ultrasonic method for the selective synthesis modificationsand support of the oxide, hydroxide and peroxide forms of Zn modified LTA zeolite (ZnO@NaA, $Zn(OH)_2$@NaA and ZnO_2@NaA).

Ultrasound can influence various stages of the synthesis process of zeolitic materials: before, during, and after crystallization. The application of sonochemical methods has gained relevance in the fields of crystallization, due to its ability to enhance and accelerate crystal formation by reducing the induction period. These improvements are essential for achieved through acoustic cavitation, where ultrasonic waves induce the collapse of bubbles that generate high-energy microenvironments, releasing free radicals that promote controlled nucleation and accelerate crystal growth. This approach, known as sonocrystallization, allows for a reduction in synthesis time, a decrease in average crystal size, improved particle size distribution, and better control over material morphology. Furthermore, the simplicity and efficiency of this method make it a promising alternative to conventional hydrothermal methods for obtaining zeolitic materials [28].

Con formato: Fuente: +Cuerpo en alfabeto complejo (Arial), Color de fuente: Negro, Inglés (Estados Unidos), Resaltar

Con formato: Fuente: +Cuerpo en alfabeto complejo (Arial), Color de fuente: Negro, Inglés (Estados Unidos), Resaltar

Con formato: Fuente: +Cuerpo en alfabeto complejo (Arial), Color de fuente: Negro, Inglés (Estados Unidos), Resaltar

Con formato: Color de fuente: Negro, Inglés (Estados Unidos), Resaltar

- In line 76, the authors state: "More recently, sonochemical approaches, such as sono-assisted precipitation (P-US), have emerged as innovative techniques for modifying zeolites." I believe this topic could be further developed. As it stands, the introduction section is weak. The authors should expand the literature review for each topic covered and include more details about the current state of the art. Based on this, clearly define the research gap and explain the limitations of previous studies in relation to your contribution. What is your novelty, specifically? While it becomes apparent throughout the manuscript, it needs to be stated more directly in the introduction to convince the reader.

Author response: We appreciate the reviewer's thoughtful feedback on the introduction section. In response, we have substantially revised this part of the manuscript to better contextualize the study. Specifically, we have:

- Expanded the literature review to include recent developments in sonochemical techniques for zeolite modification, highlighting key findings and methodologies.

- Clearly defined the existing research gap by discussing the limitations of conventional modification methods and the underexplored potential of sono-assisted approaches, particularly in the context of Zn incorporation into NaA zeolites.
- Explicitly stated the novelty of our work: the systematic use of different Zn species under sonochemical conditions to tailor the physicochemical properties of NaA zeolite, with a focus on structural, morphological, and surface charge modifications.

These improvements aim to clarify our study's contribution and strengthen the foundation of the manuscript.

Changes in text

Zinc-based species Researchers have explored Zn-based compounds such as ZnO [14–17], $Zn(OH)_2$ [18,19], and ZnO_2 [20–22] to develop advanced materials for various applications. These zinc-based nanoparticles have garnered significant attention among these nanoparticles for their catalytic, photocatalytic, and oligodynamic properties. Zinc's interactionsThe interaction of Zinc with biological systems further underscoreunderscores its relevance. The ion exchange of Zn^{2+} cations in LTAConsidering that ZnO is straightforward. Researchers have explored the incorporation of Zn-based compounds such as ZnO [14–17], $Zn(OH)_2$ [18,19], and ZnO_2 [20–22] intomain compound of interest in the literature that is often stabilized and most studied within zeolite matrices to develop advanced materials for various applications.

Post (ZnO@LTA) [23–27], we propose an ultrasonic method for the selective synthesis modificationsand support of the oxide, hydroxide and peroxide forms of Zn modified LTA zeolite (ZnO@NaA, $Zn(OH)_2$@NaA and ZnO_2@NaA).

Ultrasound can influence various stages of the synthesis process of zeolitic materials: before, during, and after crystallization. The application of sonochemical methods has gained relevance in the fields of crystallization, due to its ability to enhance and accelerate crystal formation by reducing the induction period. These improvements

Con formato: Fuente: +Cuerpo en alfabeto complejo (Arial), Inglés (Estados Unidos), Resaltar

Con formato: Fuente: +Cuerpo en alfabeto complejo (Arial), Inglés (Estados Unidos), Resaltar

Con formato: Fuente: +Cuerpo en alfabeto complejo (Arial), Inglés (Estados Unidos), Resaltar

Con formato: Fuente: +Cuerpo en alfabeto complejo (Arial), Inglés (Estados Unidos), Resaltar

Con formato: Fuente: +Cuerpo en alfabeto complejo (Arial), Inglés (Estados Unidos), Resaltar

Con formato: Fuente: +Cuerpo en alfabeto complejo (Arial), Inglés (Estados Unidos), Resaltar

Con formato: Fuente: +Cuerpo en alfabeto complejo (Arial), Inglés (Estados Unidos), Resaltar

Con formato: Fuente: +Cuerpo en alfabeto complejo (Arial), Color de fuente: Negro, Inglés (Estados Unidos), Resaltar

Con formato: Fuente: +Cuerpo en alfabeto complejo (Arial), Color de fuente: Negro, Inglés (Estados Unidos), Resaltar

Con formato: Fuente: +Cuerpo en alfabeto complejo (Arial), Color de fuente: Negro, Inglés (Estados Unidos), Resaltar

are essential for achieved through acoustic cavitation, where ultrasonic waves induce the collapse of bubbles that generate high-energy microenvironments, releasing free radicals that promote controlled nucleation and accelerate crystal growth. This approach, known as sonocrystallization, allows for a reduction in synthesis time, a decrease in average crystal size, improved particle size distribution, and better control over material morphology. Furthermore, the simplicity and efficiency of this method make it a promising alternative to conventional hydrothermal methods for obtaining zeolitic materials [28].

This ultrasonic irradiation can be used in the pre-synthesis stage, particularly during the physical mixing of different zeolite types, and has proven to be an effective method. The physical effects of ultrasound in heterogeneous media (liquid-solid) generate cavitation that leads to microbubbles' formation and collapse, producing shock waves, high temperatures, and localized high pressures. These physical effects lead to the breakup of particle agglomerates, promoting a more uniform and stable dispersion. Several studies have reported that this technique improves mixture homogeneity, increases surface area, reduces particle size, and shortens preparation time, thereby optimizing its properties. Traditional methods, likeconditions for subsequent synthesis [29].

On the other hand, ultrasound has also been employed as a post-synthesis treatment for structural modification of zeolites. Cavitation in zeolite suspensions generates additional microscopic agitation, improving local mass transfer[30]. The most commonly used post-synthesis methods for introducing metal species (conventional methods) are impregnation and co-precipitation [23][31], have been widely employed to deposit, yielding the deposition of nanoparticles on the zeolitic surface. More recently,

Con formato: Color de fuente: Negro, Inglés (Estados Unidos), Resaltar

Con formato: Color de fuente: Negro, Inglés (Estados Unidos), Resaltar

sonochemical approaches, such as Sono-assisted precipitation (P-US), have emerged as innovative techniques for modifying zeolites. This ultrasonic treatment has raisedzeolite surfaces. Nevertheless, ultrasonic irradiation has attracted attention given it promotesdue to its ability to enhance nanoparticle formation, migration, stabilization, and immobilization on the zeolite surface [24]. Moreover, these processes benefit fromzeolitic surfaces [32]. These effects are attributed to the unique conditions thatextreme localized environments generated by ultrasonic waves create, including localized high, such as elevated temperatures and pressures, which enhance the efficiency of: (i) nanoparticle synthesis and , (ii) surface deposition [25], and (iii) homogenous dispersion [33].

Nevertheless, despite the multiple documented benefits of ultrasound at various stages of zeolite modification, its specific application in incorporating metal species such as Zn into NaA-type zeolites has been scarcely explored. Traditional methods for this incorporation present significant limitations, such as poor metal dispersion and the time-consuming nature of the process[34,35]. In this context, sono-assisted techniques represent a promising yet underdeveloped strategy to overcome these limitations.

This study employs aaddresses this research gap by employing a systematic sonochemical approach to modify LTA (NaA) zeolite depositingwith different Zn species. Selectively synthesizing and dispersing through the zeolitic surface, zinc-based oxide, hydroxide and peroxide nanoparticles. Characterizing the physicochemicalThe impact of ultrasonic irradiation on the Zn incorporation, zeolite morphology, and surface properties of the resultingmaterial Zn-modified LTA composites and the role of ultrasonic irradiation in enhancing nanoparticle formation and distribution.was explored. By leveraging the extraordinaryexceptional ion-exchange capabilities of LTA zeolite, this research seeks to

Con formato: Color de fuente: Negro, Inglés (Estados Unidos), Resaltar

Con formato: Color de fuente: Negro, Inglés (Estados Unidos), Resaltar

Con formato: Color de fuente: Negro, Inglés (Estados Unidos), Resaltar

Con formato: Color de fuente: Negro, Inglés (Estados Unidos), Resaltar

Con formato: Color de fuente: Negro, Inglés (Estados Unidos), Resaltar

Con formato: Color de fuente: Negro, Inglés (Estados Unidos), Resaltar

Con formato: Color de fuente: Negro, Inglés (Estados Unidos), Resaltar

Con formato: Color de fuente: Negro, Inglés (Estados Unidos), Resaltar

Con formato: Color de fuente: Negro, Inglés (Estados Unidos), Resaltar

Con formato: Color de fuente: Negro, Inglés (Estados Unidos), Resaltar

Con formato: Color de fuente: Negro, Inglés (Estados Unidos), Resaltar

~~contribute~~contributes to the growing field of ~~nanoparticle-zeolite composites and explore their~~sonochemistry in zeolitic materials functionalized with metal nanoparticles, proposing potential applications in catalysis, bioengineering, and beyond.

.

- The methodology is clear and provides sufficient detail for reproducing the research independently—an essential aspect of scientific work. However, I encourage the authors to justify each methodological choice with appropriate citations.

Author response:

We thank the reviewer for acknowledging the clarity of the methodology section and for the valuable suggestion. In response, we have carefully reviewed the entire methodology and made several modifications to improve precision. We have also included the synthesis steps, characterization techniques, and experimental conditions. These additions aim to reinforce the scientific rigor and reproducibility of our study.

- Figure 1(a): the graph presented alongside the image is unclear. While the authors successfully conveyed their message in parts (b)–(d), the same cannot be said for part (a). Please revise and improve its clarity.

Authors response:

We appreciate the reviewer's observation regarding Figure 1(a). In response, we have revised and improved the clarity of the graph by adjusting the resolution, enhancing contrast, and ensuring that all labels, axes, and data points are clearly visible. These modifications aim to ensure that the message is conveyed as effectively as in parts (b)–(d). The updated figure has been included in the revised manuscript.

Changes in text

Con formato: Color de fuente: Negro, Inglés (Estados Unidos), Resaltar

Con formato: Color de fuente: Negro, Inglés (Estados Unidos), Resaltar

Con formato: Color de fuente: Negro, Inglés (Estados Unidos), Resaltar

Con formato: Color de fuente: Negro, Inglés (Estados Unidos), Ligaduras: Ninguna

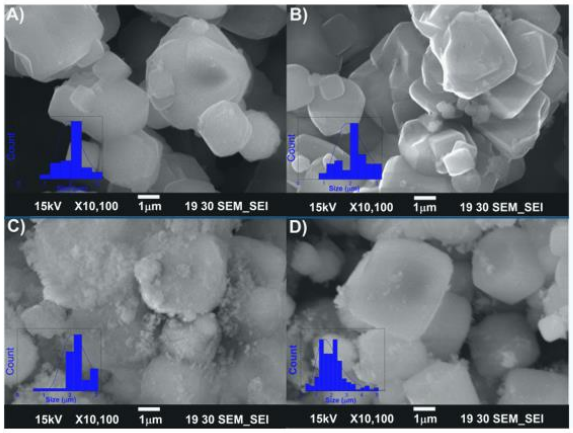

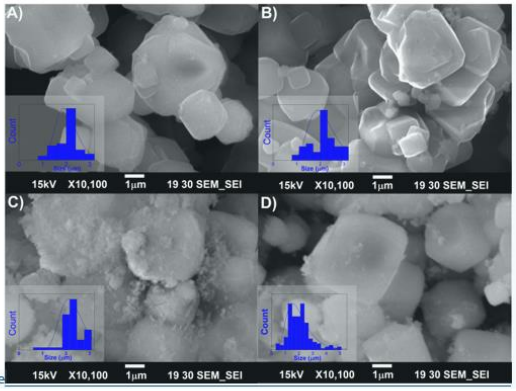

Figure

Fig 1:. Scanning electron microscopy (SEM) images). A) Micrographs of cubic NaA zeolite particles. (A) Untreated cubic NaA particles. (B) Cubic particles , untreated and after ultrasonic treatment and, under B) $Zn(OH)_2$ synthesis conditions. (, C) Cubic NaA structure surrounded by clusters of nanoparticles after

- As a general comment, I would appreciate a comparison of the efficiency of the material synthesized in this work with that of equivalent materials produced using other techniques. An analysis of energy demand versus efficiency in the context of the sonochemical treatment would greatly enhance the value of this work.

Authors Response:

We thank the reviewer for this insightful suggestion. In response, we have added a brief comparison in the Discussion section addressing the efficiency of the materials synthesized via sonochemical treatment relative to those produced by conventional methods, referencing relevant literature. Additionally, we have included a qualitative analysis of the energy demand of the sonochemical

process in relation to its observed enhancements in material properties. This provides a broader context for evaluating the benefits of the sono-assisted approach.

Changes in text

Additionally, when comparing the overall efficiency of the sono-assisted methods to traditional approaches, it becomes evident that sonochemistry offers a more energy-conscious alternative. The use of ultrasonic energy enables rapid nucleation and controlled growth of particles at moderate temperatures (65 °C) and short synthesis times (≤30 min), avoiding prolonged heating cycles required in conventional methods, such as calcination (typically >400 °C for several hours) or hydrothermal synthesis (>100 °C for >12 h) [114,117]. These characteristics significantly reduce the energy input per unit of material obtained, especially when integrated with in-situ deposition strategies.

In addition, the improved material performance, expressed through increased metal dispersion, narrower particle size distribution, and larger surface area, justifies the moderate power demand of ultrasonic equipment. This aligns with our previous studies where sonochemical synthesis is used as an ecological and rapid approach to species formation into zeolites [39,97,118,119].

- The conclusion section should be more quantitative. Include specific data to strengthen this section.

Authors response:

We appreciate the reviewer's suggestion to improve the conclusion by including more quantitative data. In response, we have revised the Conclusion section to incorporate specific values related to key findings—such as changes in surface area and cation exchange, highlighting the impact of zinc incorporation and sonochemical treatment by the different concentrations and pH values used. These additions aim to strengthen the summary and reinforce the significance of the results.

Changes in text

The synthesized LTA zeolite in its sodium form, with a Si/Al ratio of 1:1, was successfully modified with Zn species via an ultrasonic treatment. ~~The~~This sono-assisted

synthesis and support of Zn(OH)$_2$, ZnO, and ZnO$_2$ nanoparticles, under each specific ~~conditions, were synthesized and supported with~~condition, resulted in a high dispersion within the NaA zeolite, yielding three distinct ~~samples~~surface modifications, Zn(OH)$_2$@NaA, ZnO@NaA, and ZnO$_2$@NaA, respectively. The applied ultrasonic treatment did not impact the morphology of the zeolite, ~~effectively depositing the desired zinc species within the NaA structure. However, further studies are needed to increase the concentration of the deposited Zn species. It is challenging to selectively synthesize Zn(OH)$_2$ or ZnO$_2$ nanoparticles without obtaining the competing ZnO phase.~~nor did it compromise the crystallinity of the LTA structure. However, for sample Zn(OH)$_2$@NaA, the changes were not significant.

Con formato: Inglés (Estados Unidos), Sin Superíndice / Subíndice , Resaltar

~~The~~In contrast, in samples ZnO@NaA and ZnO$_2$@NaA, from the refinement data, the introduced Zn~~-modified zeolites~~ species were localized at cation exchange site I and in the α-cage, causing a cell contraction from 24.6 Å to 24.4 Å. These results correlated with the values obtained by ICP-OES and EDS, detecting Zn^{2+} in ~50% of cation exchange sites. Where the introduced ZnO or ZnO$_2$ species significantly enhanced the surface area (>400 m^2 g^{-1}) and pore volume (>0.200 cm^3 g^{-1}).

These findings suggest that the interaction of Zn species is not merely superficial but is involved in modifying the pore network of the zeolite, effectively depositing the desired zinc species within the NaA structure. This is exhibited ~~a polycrystalline structure with~~ by the distortions ~~caused by~~ of NaA when modified with the different Zn species nanoparticles. ~~Where the pore size of NaA was significantly affected, increasing both pore size and surface area. Potentially enhancing the adsorption~~Given that these distortions were not detected when sonicating without Zn species precursors, the changes in the

textural and optical properties of Zn-modified NaAthe zeolitic phase were attributed to the sono-assisted Zn modification.

Further studies are needed to increase the concentration of the desired Zn species in a specific local environment and to overcome the challenging selective synthesis of $Zn(OH)_2$ or ZnO_2 nanoparticles without obtaining a mixture with the competing ZnO phase. Thus, this applied ultrasonic-assisted methodology holds potential for synthesizing and supporting different types of nanoparticles on zeolites for various applications. Given that synergistic effects occur when combining zeolites with nanoparticles, which enhances the functional properties of the resulting materials. Future research is focused on optimizing the synthesis reaction by considering ultrasonic power, temperature, and time, expanding their applicability in different fieldsintroducing new functional sites that can enhance catalytic activity and potentially confer antimicrobial properties. These modifications broaden the applicability of the zeolite, making it a promising material for both biomedical and catalytic applications.

Con formato: Fuente: +Cuerpo en alfabeto complejo (Arial), Inglés (Estados Unidos), Resaltar

Con formato: Fuente: +Cuerpo en alfabeto complejo (Arial), Inglés (Estados Unidos), Resaltar

Con formato: Fuente: +Cuerpo en alfabeto complejo (Arial), Color de fuente: Texto 1, Inglés (Estados Unidos), Resaltar

Con formato: Fuente: +Cuerpo en alfabeto complejo (Arial), Color de fuente: Texto 1, Inglés (Estados Unidos), Resaltar, Ligaduras: Estándar + Contextual

An excellent discussion on the characterization of the synthesized material was provided, which adds significant value to this manuscript. In my opinion, the manuscript can be considered for publication in PLOS ONE once these points are carefully addressed.

Authors response:

We sincerely thank the reviewer for the encouraging comments and for recognizing the value of our discussion on the characterization of the synthesized material. We have carefully addressed all the points raised and made the necessary revisions to strengthen the manuscript. We appreciate your recommendation and thoughtful review.

---

6. PLOS authors have the option to publish the peer review history of their article (what does this mean?). If published, this will include your full peer review and any attached files.

If you choose "no", your identity will remain anonymous but your review may still be made public.

Do you want your identity to be public for this peer review? For information about this choice, including consent withdrawal, please see our Privacy Policy.

Reviewer #1: No

Reviewer #2: No

Reviewer #3: **Yes:** Tomislav Tosti

Reviewer #4: No

Reviewer #5: No

[NOTE: If reviewer comments were submitted as an attachment file, they will be attached to this email and accessible via the submission site. Please log into your account, locate the manuscript record, and check for the action link "View Attachments". If this link does not appear, there are no attachment files.]

While revising your submission, please upload your figure files to the Preflight Analysis and Conversion Engine (PACE) digital diagnostic tool, https://pacev2.apexcovantage.com/. PACE helps ensure that figures meet PLOS requirements. To use PACE, you must first register as a user. Registration is free. Then, login and navigate to the UPLOAD tab, where you will find detailed instructions on how to use the tool. If you encounter any issues or have any questions when using PACE, please email PLOS at figures@plos.org. Please note that Supporting Information files do not need this step.

In compliance with data protection regulations, you may request that we remove your personal registration details at any time. (Remove my information/details). Please contact the publication office if you have any questions.
